# Short-term inhibition of 11β-hydroxysteroid dehydrogenase type 1 reversibly improves spatial memory but persistently impairs contextual fear memory in aged mice

**DOI:** 10.1016/j.neuropharm.2014.12.005

**Published:** 2015-04

**Authors:** Nicola Wheelan, Scott P. Webster, Christopher J. Kenyon, Sarah Caughey, Brian R. Walker, Megan C. Holmes, Jonathan R. Seckl, Joyce L.W. Yau

**Affiliations:** a Centre for Cognitive Aging and Cognitive Epidemiology, University of Edinburgh, UK; b Endocrinology Unit, BHF Centre for Cardiovascular Science, University of Edinburgh, UK

**Keywords:** Hippocampus, Corticosterone, Spatial memory, Fear conditioning, Y-maze

## Abstract

High glucocorticoid levels induced by stress enhance the memory of fearful events and may contribute to the development of anxiety and posttraumatic stress disorder. In contrast, elevated glucocorticoids associated with ageing impair spatial memory. We have previously shown that pharmacological inhibition of the intracellular glucocorticoid-amplifying enzyme 11β-hydroxysteroid dehydrogenase type 1 (11β-HSD1) improves spatial memory in aged mice. However, it is not known whether inhibition of 11β-HSD1 will have any beneficial effects on contextual fear memories in aged mice. Here, we examined the effects of UE2316, a selective 11β-HSD1 inhibitor which accesses the brain, on both spatial and contextual fear memories in aged mice using a vehicle-controlled crossover study design.

Short-term UE2316 treatment improved spatial memory in aged mice, an effect which was reversed when UE2316 was substituted with vehicle. In contrast, contextual fear memory induced by foot-shock conditioning was significantly reduced by UE2316 in a non-reversible manner. When the order of treatment was reversed following extinction of the original fear memory, and a second foot-shock conditioning was given in a novel context, UE2316 treated aged mice (previously on vehicle) now showed increased fear memory compared to vehicle-treated aged mice (previously on UE2316). Renewal of the original extinguished fear memory triggered by exposure to a new environmental context may explain these effects. Thus 11β-HSD1 inhibition reverses spatial memory impairments with ageing while reducing the strength and persistence of new contextual fear memories. Potentially this could help prevent anxiety-related disorders in vulnerable elderly individuals.

## Highlights

• Aged mice were treated with UE2316 using a vehicle-controlled crossover design.
• Short-term UE2316 treatment improves spatial memory in a reversible manner.
• Contextual fear memory retention was impaired with UE2316.
• Contextual fear memory effects persisted following reversal of treatment.

## 1 Introduction

Prolonged exposure to elevated glucocorticoids as a consequence of increased hypothalamic pituitary adrenal (HPA) axis activity during ageing associates with impaired hippocampal synaptic plasticity and spatial memory decline in rodents (Issa et al., 1990; VanGuilder et al., 2011) and in humans (Dodt et al., 1994; Lupien et al., 1998). Evidence, mainly from animal studies, suggest that age-related spatial memory impairments result from the cumulative effects of elevated glucocorticoid levels on brain structure rather than on any acute effects of high glucocorticoids (Sapolsky, 1987; Seckl and Olsson, 1995). Indeed, rats are protected against age-related memory impairments when circulating glucocorticoids are maintained at low levels throughout life such as adrenalectomy with low dose corticosterone (CORT) replacement or by neonatal programming (Landfield et al., 1981; Meaney et al., 1988).

Glucocorticoids readily diffuse into the brain from the circulation to activate high affinity mineralocorticoid receptors (MR) and lower affinity glucocorticoid receptors (GR). Both MR and GR are expressed in limbic regions involved in cognitive function (de Kloet et al., 1999; Oitzl and de Kloet, 1992). Tissue glucocorticoid levels are also influenced, however, by 11β-hydroxysteroid dehydrogenase type 1 (11β-HSD1), an intracellular glucocorticoid-amplifying enzyme which regenerates active glucocorticoids (corticosterone in rodents; cortisol in humans) from circulating inert forms (11-dehydrocorticosterone, cortisone) (Wyrwoll et al., 2011). 11β-HSD1 is highly expressed within the hippocampus, amygdala, cortex and other CNS regions underpinning cognition (Moisan et al., 1990; Pelletier et al., 2007; Sandeep et al., 2004).

Glucocorticoids locally generated by 11β-HSD1 play a major role in age-related cognitive impairments (Maclullich et al., 2010; Yau and Seckl, 2012). Thus mice with lifelong deficiency of 11β-HSD1 resist age-dependent spatial memory impairments even though plasma CORT levels are elevated to the same level as wildtype mice (Yau et al., 2007, 2001). Conversely, transgenic mice with modest forebrain-specific overexpression of 11β-HSD1 show accelerated memory deficits with ageing (Holmes et al., 2010). Moreover, we have shown that just 10 days treatment with a selective 11β-HSD1 inhibitor improves spatial memory in aged mice without affecting plasma CORT levels (Sooy et al., 2010).

Although elevated glucocorticoids or stress can impair the retrieval of spatial memories (de Quervain et al., 1998), they also strengthen aversive/emotional memories (Roozendaal and McGaugh, 2011; Roozendaal et al., 2006). The hippocampus and amygdala play dissociable roles in processing the contextual and emotional properties of a fear memory while the prefrontal cortex integrates the two (Phillips and LeDoux, 1992; Zelikowsky et al., 2014). Contextual fear memory is enhanced by glucocorticoids in a dose-dependent manner (Abercrombie et al., 2003; Okuda et al., 2004; Roozendaal and McGaugh, 2011) and is impaired by adrenalectomy (Pugh et al., 1997).

Fear learning is highly adaptive and evolutionarily conserved to protect individuals against repeated danger. But fear memories can sometimes persist well beyond the traumatic experience. This can predispose some individuals to anxiety-related disorders including post-traumatic stress disorder (PTSD). One common feature of PTSD is exaggerated and inappropriate fear responses. Moreover, recent evidence in war veterans suggests that PTSD is associated with a higher risk of dementia (Meziab et al., 2014).

While 11β-HSD1 inhibition is beneficial in attenuating age-related spatial memory decline, it is not known if 11β-HSD1 is involved in glucocorticoid-associated emotional/fear memories, notably with ageing. We therefore examined the effects of short-term inhibition of 11β-HSD1 on spatial and contextual fear memories in aged C57BL/6J mice using a vehicle controlled cross-over design to test whether such effects are reversible.

## 2 Materials and methods

### 2.1 Animals

Male C57BL/6J mice were bred in-house and maintained in groups of 3–5 per cage on a 12 h light/dark cycle (lights on at 07:00 A.M.) with food and water *ab libitum* until experimentation at 24 months old. All procedures and behavioural experiments were performed between 8.00 and 11.30 a.m. and approved by the local University of Edinburgh animal welfare ethical review body and performed in strict accordance with the U.K. Animals (Scientific Procedures) Act, 1986.

### 2.2 Y-maze

Spatial memory was assessed in a two trial Y-maze task as described (Sooy et al., 2010). The time spent in the novel arm was calculated as a percentage of total time in all three arms. A 1 min inter-trail interval (ITI) was used to control for spontaneous novelty exploration and to assess vision and a 2 h ITI was used to assess hippocampal-dependent spatial recognition memory.

### 2.3 Contextual fear conditioning

The fear conditioning (FC) apparatus (Coulbourn Instruments, Whitehall PA) consisted of an animal enclosure (25 cm × 25 cm × 38 cm) made up of two inter-changeable aluminium side walls and Plexiglas rear and front walls with a removable shock grid floor of stainless steel rods (3.2 mm diameter, 4.7 mm apart), all housed within a sound-attenuating chamber illuminated with a single house light. The grid floor was connected to a precision-regulated shocker that delivered the electric footshock stimuli controlled by FreezeFrame software (Actimetrics). A camera located on top of the FC enclosure recorded the activity of the mice. Freezing behaviour (complete absence of movement except for breathing) was analysed using FreezeFrame software (Actimetrics). The FC enclosure was cleaned with 70% ethanol and air-dried before each trial and between mice.

Each mouse was habituated to the FC enclosure within a neutral context (silver aluminium tiles alongside the walls with no visual spatial cues or scents) for 4 min on two consecutive days. On day 1 of training (after habituation), mice were placed in the FC enclosure within an enriched context (aluminium tiles swapped for black and white tiles placed in unique pattern with addition of vanilla or almond essence odours) for 3 min of acclimatization. Mice were then given a context conditioned stimulus (CS, FC enclosure context) paired with an unconditioned stimulus [US, two electric foot shock(s) separated by a 30 s interval (2 s, 0.6 mA)]. Contextual fear memory of the foot shock was assessed 24 h later when mice were exposed to the same training context but without any shocks. Freezing responses were measured for 240s in the FC enclosure. Extinction trials followed the same protocol as the retention trials where the mice were re-exposed to the CS without reinforcement of the US.

### 2.4 11β-HSD1 activity

Hippocampal and cortex tissues were homogenised and assayed for 11-ketosteroid reductase activity as described previously (Sooy et al., 2010).

### 2.5 Corticosterone radioimmunoassay (RIA)

Plasma corticosterone levels were measured using an in-house RIA (Al Dujaili et al., 1981) modified for microtiter plate scintillation proximity assay (GE Healthcare, UK). The intra-assay and inter-assay coefficients of variation were 9.4% and 9.2%, respectively.

### 2.6 UE2316

The novel 11β-HSD1 inhibitor (UE2316; [4-(2-chlorophenyl-4-fluoro-1-piperidinyl][5-(1H-pyrazol-4-yl)-3-thienyl]-methanone) was synthesised by High Force Ltd, UK according to methods previously described (Webster et al., 2011).

### 2.7 Experimental schedule

**Fig. 1:**
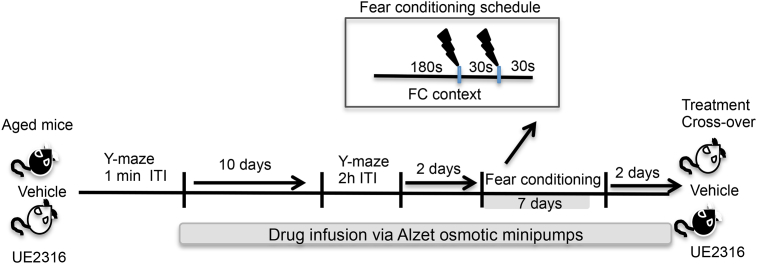
Experimental schedule for the effects of UE2316 treatment on spatial and contextual fear memory in a vehicle controlled cross over design. Prior to behavioural testing (Y-maze (2 h inter-trial interval (ITI)) and fear conditioning (FC)), 24 m aged mice were treated via subcutaneously implanted Alzet osmotic minipumps with UE2316 (10 mg/kg/day) or vehicle (*n* = 8–9/group).

UE2316 treatment and behavioural testing in the 24 months old C57BL/6J mice were carried out as shown in Fig. 1. All aged C57BL/6J mice were first tested in the Y-maze (1 min ITI) to confirm intact vision and preferential exploration of the novel arm before random selection for either UE2316 (10 mg/kg/day) or vehicle (50% dimethyl sulfoxide: 50% polyethylene glycol) treatment via two osmotic minipumps (5 mg/kg/day per pump; model 2004: nominal pump rate 0.25 μl/h; Alzet, USA) implanted subcutaneously under anaesthesia (Isoflurane 3%). Spatial memory was re-assessed in the Y-maze (2 h ITI) on the 10th day of drug treatment. Four days later, whilst still receiving drug treatment, all mice underwent contextual fear conditioning as described above followed 24 h later by context retention and then three extinction trials conducted over the course of 5 days. On the morning following the final extinction trial, tail venesection blood samples were taken for CORT measurements. Then, on the following day, the treatment groups were crossed over such that UE2316 pumps were replaced with vehicle pumps and vice versa. After ten days drug treatment, spatial memory and the retention and extinction of contextual fear memory were assessed as before, but with different visual spatial cues around the Y-maze and a new context in the FC enclosure. Again, tail venesection blood samples were taken the morning following the final extinction trial. All mice were culled the following morning by cervical dislocation and brains dissected, frozen on dry ice and stored at −80 °C for 11β-HSD1 activity assays.

### 2.8 Statistical analysis

Plasma CORT levels and 11β-HSD1 activity (% conversion of [^3^H]-11DHC to [^3^H]-CORT) were analysed by Student's unpaired *t*-tests for between treatment group comparisons. Y-maze data were analysed by two-way ANOVA, with treatment and time in arms as the independent variables. Within animal comparisons of % time in arms of Y-maze were analysed by multiple paired *t*-tests with Bonferroni adjusted *P* value. Contextual fear memory training, retention and extinction data were analysed by two-way ANOVA, with footshock or days and treatment as the independent factors. Statistical significance was determined post-hoc by multiple *t*-tests using the Holm-Sidak method, with alpha = 5%. All data were analysed using GraphPad Prism 6 software. Significance was set at *p* < 0.05. Data are expressed as means ± SEM.

## 3 Results

### 3.1 Plasma CORT levels are unaltered with 11β-HSD1 inhibition

**Fig. 2:**
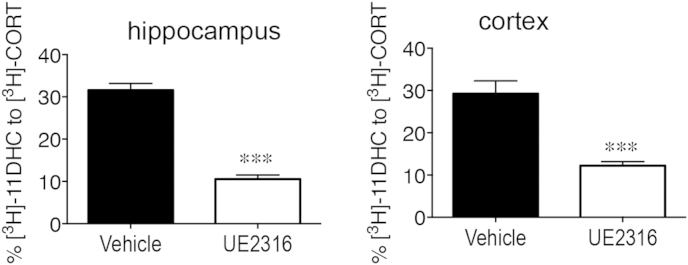
UE2316 reduces 11β-HSD1 activity in the aged mouse brain. 11β-HSD1 activity measured ex-vivo as % conversion of [^3^H]-11dehydrocorticosterone to [^3^H]-corticosterone in hippocampus and cortex from UE2316 and vehicle treated aged mice. Results represent mean ± SEM. ***P < 0.001 vs vehicle group.

UE2316 treatment significantly reduced 11β-reductase activity in the hippocampus and cortex of the aged mice measured ex vivo (65% and 58% decrease in hippocampus and cortex respectively (*P* < 0.001 compared to vehicle controls), Fig. 2). Plasma CORT levels were not significantly affected by drug treatment [initial treatment: vehicle (33.81 ± 6.99 nM, *n* = 8) and UE2316 (17.25 ± 5.43 nM, *n* = 9); cross-over treatment: vehicle (39.7 ± 8.07 nM, *n* = 8) and UE2316 (23.96 ± 4.48 nM, *n* = 8)].

### 3.2 UE2316 reversibly improves spatial memory in aged mice

**Fig. 3:**
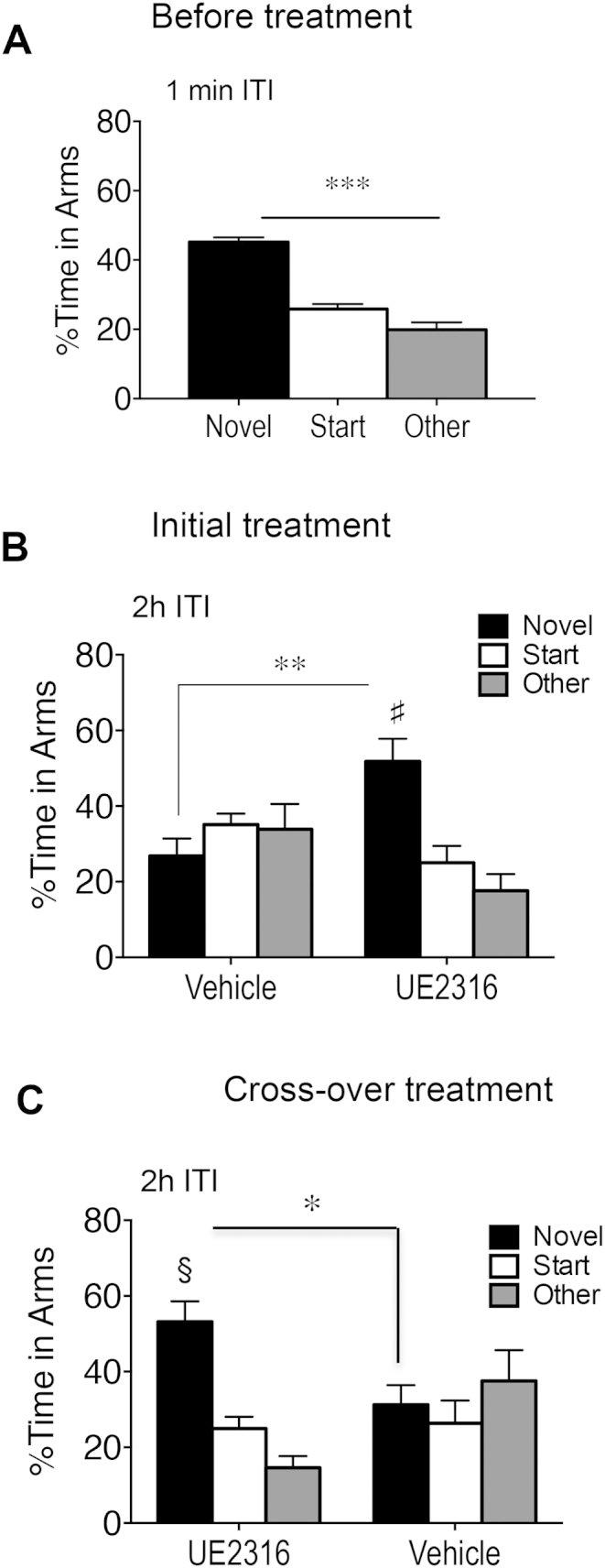
Short-term UE2316 treatment reversibly improves spatial memory in aged C57BL/6J mice. (A) All aged mice were tested in the immediate version of the Y-maze (1 min inter-trial interval (ITI)) confirming preferential exploration of the novel arm and no visual problems identifying cues around the maze. (B) UE2316 improved spatial memory (more time spent in novel arm) on the 10th day of treatment while vehicle treated aged mice remained impaired in the Y-maze (*n* = 8–9/group). (C) Improved spatial memory in UE2316 treated mice reversed after swapping to vehicle, while impaired spatial memory in vehicle-treated mice improved after swapping to UE2316. Results represent mean ± SEM. ***P < 0.0001, §*P* < 0.01 and ♯*P* < 0.05 vs. other arms. *P < 0.05 vs corresponding treatment.

Prior to UE2316 drug treatment, all 24 m aged mice tested in the immediate version of the Y-maze [1 min inter-trial interval (ITI)], spent more time in the novel arm (*P* < 0.0001, compared to previously visited arms, Fig. 3A), confirming neophilia (normal response to the novel arm) and showing intact vision. Using the 2 h ITI to test spatial memory retention, the resulting data showed a significant interaction of UE2316 treatment and % time in arms (*F*_2,45_ = 9.8, *P* < 0.001 and *F*_2,45_ = 3.82, *P* < 0.05, respectively). Vehicle control aged mice were impaired failing to spend more time in novel arm (Fig. 3B). However, UE2316 treated aged mice showed improved spatial memory on the 10th day of treatment (more time in novel arm than previously visited arms (*p* < 0.01); time in novel arm was greater than in vehicle controls (*P* < 0.01, unpaired *t*-test), Fig. 3B).

To test whether the improved spatial memory persists when UE2316 treatment stops, the osmotic minipumps were removed and replaced with new pumps filled with UE2316 or vehicle. When re-tested in the Y-maze (2 h ITI) on the 10th day of treatment, there was a significant interaction of UE2316 treatment and % time in arms (*F*_2,45_ = 8.0, *P* < 0.01 and *F*_2,45_ = 5.69, *P* < 0.01, respectively). Aged mice with treatment changed from UE2316 to vehicle, reverted to impaired spatial memory (similar times in all three arms; % time in novel arm was significantly lower on vehicle than when previously on UE2316 treatment (*P* < 0.05, paired *t*-test, Fig. 3B and C). Conversely, aged mice with treatment changed from vehicle to UE2316 now showed improved spatial memory (% time in novel arm greater than previously visited arms, *P* < 0.01); time in novel arm was greater than in vehicle controls (*P* < 0.05, unpaired *t*-test, Fig. 3C) and greater than novel arm when previously on vehicle, (*P* < 0.05, paired *t*-test, Fig. 3B and C).

### 3.3 UE2316 impairs contextual fear memory retention in aged mice with effects persisting after drug withdrawal

**Fig. 4:**
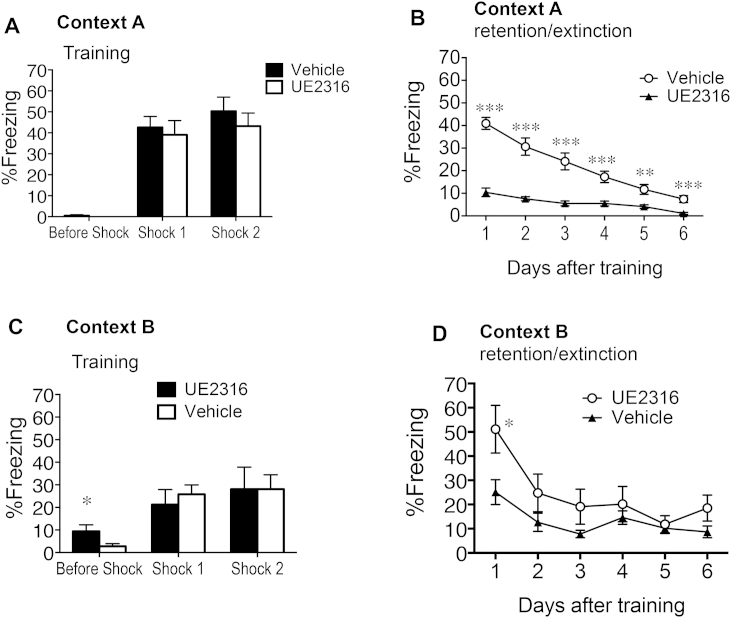
Short-term UE2316 treatment impairs contextual fear memory in aged C57BL/6J mice with effects persisting after drug withdrawal. (A) When exposed to training in context A, no freezing was observed prior to foot shocks but after each foot shock, all mice showed moderate levels of freezing which were not affected by UE2316 treatment. (B) UE2316 treatment reduced contextual fear memory retention compared to vehicle controls for each consecutive day following training. Level of freezing declined with each consecutive day of re-exposure to context A. (C) When treatments were swapped over mice now showed low levels of freezing when exposed to training context B, even prior to the foot shocks. UE2316 did not affect the level of freezing after each foot shock. (D) UE2316 (previously vehicle) treatment now enhanced contextual fear memory retention 1 day following training. Level of freezing declined with each consecutive day of re-exposure to context B. Results represent mean ± SEM. ***P < 0.001, **P < 0.01, *P < 0.05 vs corresponding treatment group.

To examine whether contextual fear memory is affected by UE2316 treatment, aged mice were tested in the fear conditioning apparatus following the Y-maze. During training in context A, neither UE2316 nor vehicle treated animals displayed any freezing prior to the footshocks but similar freezing responses were shown immediately after the footshocks (>35%, Fig. 4A). UE2316 treated aged mice exhibited strikingly lower levels of freezing when re-exposed to context A for each of the 6 consecutive days after training (*F*_1,90_ = 184.5, *P* < 0.0001, Fig. 4B). Though both groups given repeated re-exposure to context A over 6 days gradually extinguished the fear memory, responses were lower in UE2316-treated mice on each occasion (*F*_5,90_ = 27.89, *P* < 0.0001, Fig. 4B) and only UE2316 treated mice fully extinguished the freezing response.

In contrast to spatial memory, the effects of UE2316 on contextual fear memory were not reversed when drug treatments were swapped. Aged mice previously on vehicle but now on UE2316 exhibited low levels of freezing to the new context B (*P* < 0.05, greater than vehicle controls), suggesting “renewal” of the original fear memory which had not fully extinguished (Fig. 4C). In contrast, aged mice which previously received UE2316 but now on vehicle treatment showed minimal baseline freezing to context B (Fig. 4C). As before, both treatment groups responded with similar freezing responses to the footshocks. However, one day after training, aged mice with treatment changed from UE2316 to vehicle showed persisting reduced contextual fear memory retention (*P* < 0.05) compared with animals now receiving UE2316 having changed from vehicle (Fig. 4D). The levels of freezing behaviour were gradually extinguished over subsequent days of re-exposure to context B with no statistically significant difference between the groups by the second day onwards (treatment, *F*_1,90_ = 13.06, *P* < 0.001; days, *F*_5,90_ = 6.98, *P* < 0.0001, Fig. 4D).

The freezing responses in aged mice on vehicle (but previously on UE2316) were lower during the initial 3 days of re-exposure to the new context B compared to the freezing responses of these mice when initially on vehicle exposed to context A (*F*_1,90_ = 25.08, *P* < 0.0001) (Fig. 4D and B). This suggests the initial UE2316 treatment effects may have persisted for some time following the swap to vehicle and affected the new fear memory. The freezing responses of aged mice on UE2316 (but previously on vehicle) were greater when exposed to context B than in the initial UE2316 treated aged mice exposed to context A (*F*_1,90_ = 44.62, *P* < 0.0001) (Fig. 4D and B). This suggests that the initial fear memory in aged mice on vehicle may have reactivated upon re-exposure to context B, and not been suppressed by current UE2316 treatment.

## 4 Discussion

In this study we provide evidence that inhibition of 11β-HSD1 differentially modulates spatial and fear memories in aged mice, with improvements in spatial memory, confirming the results with another 11β-HSD1 inhibitor, UE1961 (Sooy et al., 2010), but impairments in contextual fear memory.

In the absence of changes in plasma CORT levels, UE2316 inhibition of 11β-HSD1 to lower intracellular CORT levels in brain regions important for cognition may explain the improved memory in the aged mice. Indeed, we recently found that 10 days of UE2316 treatment reduced the dynamic rise in intrahippocampal CORT levels during memory retrieval in a Y-maze, an effect associated with improved spatial memory retention (Yau et al., in press). The rapid reversibility of the spatial memory deficits on withdrawal of UE2316 in already aged mice suggests that the impairing effects of elevated glucocorticoids on memory are not wholly attributable to irreversible effects on brain structure.

In contrast to spatial memory, the effects of glucocorticoids on fear memory are more long lasting. Previous studies have shown that glucocorticoids dose-dependently enhance the consolidation of fear memories (Abercrombie et al., 2003; Roozendaal and McGaugh, 2011) and that GR antagonists infused directly into the basolateral amygdala impairs this consolidation process (Donley et al., 2005). Thus, whereas age-related spatial memory deficits associate with elevated plasma glucocorticoids activating GRs (Yau et al., 2011), enhanced fear/emotional memories also require GRs, particularly in the amygdala (Kolber et al., 2008; Nikzad et al., 2011). Indeed, chronic restraint stress, which increases plasma CORT levels, impairs spatial memory (Luine et al., 1994) but enhances contextual fear conditioning (Conrad et al., 1999). Furthermore, during emotional situations when circulating glucocorticoid levels are elevated, the hippocampus via its interaction with the amygdala is thought to contribute contextual information associated with the fearful event (Richter-Levin and Akirav, 2000). A similar high level of plasma glucocorticoids would however impair spatial memory in a separate situation not involving a fearful event. Accordingly, reducing local tissue CORT levels in stressed or aged animals via 11β-HSD1 deficiency or inhibition would be predicted to have the opposite effect of improving spatial memory and impairing contextual fear memory. In support, even a single injection of UE2316, which would transiently lower hippocampal CORT levels has recently been shown to impair contextual fear conditioning in young adult mice (Sarabdjitsingh et al., 2014). In the current study, 10 days of UE2316 treatment in aged mice also impaired contextual fear memory retention. Ageing *per se* has not been shown to affect the retention of contextual fear memories shortly after training (within 10 days) (Bergado et al., 2011; Gould and Feiro, 2005; Oler and Markus, 1998) although one study found decreased retention of the conditioning context when tested 52 days after training in aged rats (Oler and Markus, 1998). Since activation of GRs located in the hippocampus is necessary for contextual fear memory consolidation (Abrari et al., 2009), lowered intrahippocampal CORT levels associated with UE2316 treatment (Yau et al., in press), may in part underlie the impaired contextual fear memory in the aged mice. Furthermore, it is likely that UE2316 reduced CORT levels in other brain regions involved in contextual fear memory (Orsini et al., 2013; Zelikowsky et al., 2014). Although the amygdala was not dissected for measurement of 11β-HSD1 inhibition by UE2316 in the present study, expression of 11β-HSD1 (Pelletier et al., 2007) and detectable levels of UE2316 after a single peripheral dose (Cobice et al., 2013) have been located in this brain region.

In contrast to the reversible effects of UE2316 on spatial memory in the aged mice, the impairing effects of UE2316 on contextual fear memory was not rapidly reversed when drug and vehicle treatments were swapped following extinction of the initial fear memory. This is not surprising since it is well established that, although fear memories can be extinguished by repeated exposure to the context or cue that associates with the threat, the original fear memory is not permanently erased (Zelikowsky et al., 2012). The process of extinction is itself a new learning that inhibits the expression of old learning (e.g. learning to no longer fear a previously feared situation or object, usually by repeated exposure to reminders of the original fear in a safe environment) (Quirk, 2002; Quirk and Mueller, 2008). Aged mice on UE2316 but previously on vehicle exhibited a significant amount of freezing upon exposure to a novel context consistent with “renewal” of the original extinguished fear (Orsini et al., 2013; Zelikowsky et al., 2012). Although the new context was notably different (wall tiles, spatial objects and odour), the floor grid used to deliver the shock was the same. Thus the floor grid may itself act as a reminder that renews the original fear memory, especially if memory of the initial context-shock association was particularly strong or had not fully extinguished. Surprisingly, the impairing effect of UE2316 on contextual fear memory appear to persist after treatment stops so that memory of a subsequent foot-shock conditioning in new context was less severe. How this occurs is unclear and further investigations are necessary to establish how long the effect persists. Similar findings were observed in a human study when metyrapone, used to decrease glucocorticoid levels, reduced the strength of an emotional memory trace in a long-lasting manner with effects still observed 4 days after drug administration (Marin et al., 2011).

Although UE2316 in aged mice appeared to cause an exaggerated memory response to a second round of fear conditioning in new context, this is still likely to be less than if mice had been exposed to two consecutive rounds of fear conditioning in different contexts without any protection from UE2316 treatment. This is because, in addition to renewal of the original fear memory (Orsini et al., 2013; Zelikowsky et al., 2012) in vehicle-treated aged mice, there will also be the additional retrieval of the new fear memory during vehicle or UE2316 treatment, with the latter being substantially impaired. Consistent with this hypothesis, we have recently found that when young wildtype mice are fear conditioned for a second time in a new context (one week after the first contextual fear conditioning test) there is even greater fear memory retention, whereas 11β-HSD1 deficient mice continued to show similar reduced fear memory retention upon exposure to each training context (Wheelan & Yau, unpublished).

In conclusion, spatial memory deficits in aged mice can be reversed by short-term UE2316 treatment but once treatment stops the impaired memory returns. Contextual fear memories on the other hand are impaired by UE2316 in the same aged mice but these effects are not reversed acutely when treatment is discontinued because renewal of the original extinguished fear memory in the novel context affects the new fear memory. Selective 11β-HSD1 inhibitors may not only protect against or reverse age-related spatial memory deficits but could also have the added benefit of reducing the strength of traumatic memories in vulnerable individuals.

## Disclosure statement

SPW, JRS and BRW are inventors on licenced patents owned by the University of Edinburgh covering UE2316 and related compounds, and have consulted widely for companies developing 11β-HSD1 inhibitors.
